# Emotion Regulation, Parenting, and Psychopathology: A Systematic Review

**DOI:** 10.1007/s10567-023-00452-5

**Published:** 2023-09-13

**Authors:** Jana Zitzmann, Larissa Rombold-George, Charlotte Rosenbach, Babette Renneberg

**Affiliations:** Department of Clinical Psychology and Psychotherapy, Freie Universität Berlin, Habelschwerdter Allee 45, 14195 Berlin, Germany

**Keywords:** Parents, Psychopathology, Mental disorders, Emotion regulation, Parenting, Emotion socialization

## Abstract

The presence of a parental mental disorder can lead to adverse outcomes for children. Difficulties in emotion regulation are observed across a range of mental health problems and may play a crucial role in this context. Following PRISMA guidelines, we systematically searched Medline, PsycINFO, Embase, and Web of Science for studies examining the association between emotion regulation in parents with psychopathology at a clinical or subclinical level and their parenting. The protocol was registered with the PROSPERO international prospective register of systematic reviews (CRD42021224954; January 2021). A total of 23 studies were included in the qualitative synthesis. Emotion regulation was predominantly assessed using self-report on the general ability (e.g., Difficulties in Emotion Regulation Scale). The assessment of parenting encompassed a broad range of aspects and operationalizations. Across psychopathology in parents, several aspects of difficulties in emotion regulation were associated with unfavorable emotion socialization, more negative parenting, and partially with less positive parenting. Slightly different effects were observed for posttraumatic stress disorder and anxiety disorders. For parents with depressive disorders, specific emotion regulation strategies (suppression, reappraisal) seem to buffer against negative parenting. Since the majority of studies refer only to mothers, generalization to fathers is limited. Furthermore, conclusions are limited due to study heterogeneity and lack of prospective studies. Nevertheless, findings suggest that interventions should target the improvement of emotion regulation in parents with psychopathology.

**Supplementary Information:**

The online version contains supplementary material available at 10.1007/s10567-023-00452-5.

## Background

Emotion regulation is an integral part of our daily lives and thus also plays a central role in parenting. Effective parenting involves managing one’s emotions to satisfy the needs of children and promote their long-term ability to self-regulate (Crandall et al., 2015; Dix, 1991; Rutherford et al., 2015). At the same time, the daily demands of parenting and adjusting to new roles can make it particularly challenging for parents to regulate their emotions, particularly in response to stress and demanding child behavior (Crandall et al., 2015).

Despite the importance of such factors contributing to parenting behavior, past research investigated predominantly how parenting behavior affects children. These studies provided robust evidence that positive parenting behavior such as sharing positive affect or parental warmth can promote children’s development (Berg-Nielsen et al., 2002). On the negative side, parental negativity and harsh discipline practices were identified as risk factors for negative child developmental outcomes (Berg-Nielsen et al., 2002; Dix, 1991). However, to prevent negative outcomes and promote a functional parent child relation, it is important to investigate the question what factors contribute to a specific parenting style. Accordingly, there is a growing number of empirical studies examining different factors that may influence parenting behavior, including a parent’s ability to regulate their emotions. Reviews of these studies conclude that parents who experience greater difficulty in emotion regulation tend to be more hostile and less warm toward their children. They also tend to engage more in negative parenting practices and respond with non-supportive behaviors such as minimizing or punishment to their children’s negative feelings (Barros et al., 2015; Crandall et al., 2015; Zimmer-Gembeck et al., 2022). In comparison, parents who report higher emotion regulation skills are more likely to show positive parenting behaviors (Crandall et al., 2015; Zimmer-Gembeck et al., 2022). Since children’s emotion regulation develops through interaction with their parents, it is suggested that parental emotion regulation and parenting might function as central mechanisms for transmitting self-regulation abilities across generations (Bridgett et al., 2015; Rutherford et al., 2015; Ulrich & Petermann, 2017; Zimmer-Gembeck et al., 2022). Therefore, to promote healthy child development, it is important to focus on parenting and especially parental emotion regulation as a potential central determining factor.

Most parents with chronic disturbances in emotion regulation tend to suffer from mental disorders. Empirical studies proved an association between emotion dysregulation and psychopathology, and several authors suggested its role as a transdiagnostic aspect of mental disorders (Aldao et al., 2010; Cludius et al., 2020; Fernandez et al., 2016; Lincoln et al., 2022). In their meta-analytic review, Aldao et al. (2010) found that maladaptive emotion regulation strategies such as rumination, avoidance, and suppression were strongly associated with psychopathology, particularly internalizing disorders. These internalizing disorders have also been focused in previous research. Additionally, Lincoln et al. (2022) emphasized that inflexible emotion regulation patterns not only predicted but also resulted from psychopathology across a range of disorders. Accordingly, the ability to regulate one’s emotions has been highlighted in models of psychopathology (e.g., for affective disorders, borderline personality disorder [BPD], anxiety disorders; Lincoln et al., 2022). This leads to the assumption that parents with mental disorders may have reduced the emotion regulation capacities, which could, in turn, result in problematic parenting behaviors. To provide appropriate help, it is essential to understand and address the challenges of this target group.

Baldwin et al. (2022) recently demonstrated the unique contribution of parental mental disorder for unfavorable developmental outcomes in offspring, such as internalizing and externalizing psychopathology. Importantly, this contribution remains independent of genetic confounding (Baldwin et al., 2022). Over the past few decades, a growing body of research has highlighted the difficulties faced by parents with different mental health conditions in their parenting, showing that childrearing can be particularly challenging for people experiencing mental health concerns and associated emotion dysregulation. Comprehensive reviews of these studies found psychopathology across a range of disorders to be related to a decreased quality of child parent interactions, including role-reversal dynamics, as well as more negative parenting practices like hostility (e.g., Dix & Meunier, 2009; Eyden et al., 2016; Steele et al., 2019). Nevertheless, there is significant variability in the parenting constructs and mental health conditions examined across these studies. Parents with personality disorders (mostly BPD) have been frequently studied and were found to engage in heightened overprotection and hostility while also showing reduced sensitivity toward their children (Eyden et al., 2016; Steele et al., 2019). Similarly, depressive symptoms in parents have also been extensively studied and have shown associations with increased negativity and reduced child orientation (Dix & Meunier, 2009). However, data on parenting behavior are more limited for other mental health conditions. Maliken and Katz (2013) reviewed studies investigating the impact of parental mental health problems and the ability of emotion regulation on the success of parenting interventions and subsequently suggested to focus on the promotion of emotion regulation skills across psychopathology in parents. In line with these findings, several studies indicate that impairments in parents’ emotion and cognitive control capacities might explain a lack of response to parent trainings (e.g., Crandall et al., 2015).

### Aims of the Review

In the last decade, research on emotion regulation in the context of childrearing showed increasing evidence that parental difficulties in emotion regulation may negatively influence parenting and family processes (Bridgett et al., 2015; Crandall et al., 2015; Ulrich & Petermann, 2017; Zimmer-Gembeck et al., 2022). We wanted to take a more fine-grained look at this relationship in the context of parents with mental health problems. To the best of our knowledge, this is the first systematic review summarizing research on emotion regulation and parenting considering parental mental health conditions. Our specific research question was: How is emotion regulation in parents with mental health problems related to parenting?

To address this question, we systematically reviewed empirical studies that investigated the association between emotion regulation in parents with psychopathology and their parenting. A pilot search yielded more studies on parents with psychopathology at a subclinical level rather than with diagnoses of mental disorders. To ensure a comprehensive search, we decided to include samples not only with diagnosed mental disorders but also with mental health problems at a subclinical level (i.e., elevated symptom levels). An additional research question was: What types of psychopathology were investigated? As we expected a wide range of terminology and conceptual definitions in the scope of parenting and emotion regulation research that is used interchangeably and inconsistently, further review questions were:

- How was emotion regulation in the context of parenting operationalized?
- Which aspects of parenting were investigated?

## Methods

This systematic review followed the recommendations of the Preferred Reporting Items for Systematic Review and Meta-Analysis (PRISMA) statement (Moher et al., 2009). The protocol was registered with the PROSPERO international prospective register of systematic reviews (CRD42021224954).

### Conceptualization of Emotion Regulation

Gross (1998) defined the emotion regulation as attempts of individuals to “influence which emotions they have, when they have them, and how they experience and express these emotions” (Gross, 1998; p. 275). In a recent review (Lincoln et al., 2022), psychopathology across various mental disorders was associated with an inflexible pattern of emotion regulation strategy use. In addition to the *modulation of emotional activation* by the use of specific strategies, the definition above encompasses the *experience and expression of emotions* as further aspects of emotion regulation, such as emotional awareness (Gratz & Roemer, 2004; Gross, 1998). Many individuals with mental disorders experience difficulties in this general ability to deal with emotions (e.g., Fernandez et al., 2016).

Concordantly to this conceptualization previously used in the context of research on parenting as well as on psychopathology, we decided to capture emotion regulation in a broader sense. In this regard, the construct of emotion regulation is conceptualized as a dispositional trait rather than a transient state. Accordingly, studies referring to different steps involved in the process of emotion regulation (e.g., awareness for emotions, experience of emotions, and strategy use) were included in this review. Emotion regulation in parents could have been assessed using self-report questionnaires (general questionnaires and questionnaires on specific emotion regulation strategies), interview data, or observational data.

### Conceptualization of Parenting

Several constructs have been used to study parenting to date. In their review on associations between parenting and child and parent psychopathology, Berg-Nielsen et al. (2002) provided a broad conceptualization: “parenting […] consists of parental everyday behaviour toward offspring including parents’ cognition, emotions and attributions toward their child, as well as parenting attitudes and values” (Berg-Nielsen et al., 2002; p. 531). The authors identified two main dimensions of dysfunctional parenting—parental negativity and various forms of ineffective discipline ﻿practices (Berg-Nielsen et al., 2002). Other studies addressed aspects of parenting that may not only adversely but also positively affect the child (e.g., parental warmth).

Based on the conceptualization by Berg-Nielsen et al. (2002) and approaches in previous research, we included studies addressing *aspects of parenting with positive or negative valence evident in behavioral, interactional and attitudinal dimensions* (e.g., parenting behavior, child abuse potential, emotion socialization) in the current review. Parenting could have been assessed using a range of assessment methods, e.g., observational data, questionnaires (self- and other-report), interview data, recorded incidents of child abuse or maltreatment.

### Literature Searches

A comprehensive search for relevant published and unpublished studies was conducted. The databases systematically searched were Medline (PubMed), PsycINFO (EBSCOhost), Embase (Ovid) and Web of Science. Systematic searches were conducted in December 2020 and re-run in January 2023. The search strategy was built upon the PEO (Population, Exposure, Outcomes) framework used to specify our research question: We searched for studies investigating parents (P). To guarantee a comprehensive search, we refrained from including specific terms on psychopathology. In a later step, we included only those studies that assessed psychopathology in parents via our formulated inclusion and exclusion criteria. Furthermore, studies had to provide data on emotion regulation in parents (E) along with any aspect of parenting (O) regardless of whether these constructs were a central focus of the study.

According to this framework, we used the following search terms and keywords: (parent* OR mother* OR father* OR caregiver*) AND (emotion* *regulat* OR affect* *regulat*) AND (parenting OR parent* behavi* OR parent* discipline practice* OR parent* negativity OR parent* warmth OR parent* control OR parent* hostility OR parent* sensitivity OR emotion* socialization OR child abus* OR child neglect* OR child maltreat* OR (parent AND child AND relation*) OR (parent AND child AND interact*) OR (parent AND child AND communicat*)). Search strategies for each database can be found in the appendix (see Appendix A). We identified additional records through other sources (e.g., key journals, reference lists of eligible studies and review articles, cited articles, and related articles). Additionally, we screened ClinicalTrials.gov for unpublished studies.

### Inclusion and Exclusion Criteria for the Study

We included observational studies (cohort studies, case–control studies, cross-sectional studies) and experimental studies that reported at least one cross-sectional or longitudinal relationship between emotion regulation in parents and their parenting. We further included studies with between-group comparisons contrasting individuals with high emotion regulation difficulties to controls with low emotion regulation difficulties on measures of their parenting and vice versa. Intervention studies were included only if baseline data were available. The studies had to provide a quantitative measure of emotion regulation and parenting according to the descriptions above. According to the defined characteristics of parents (mothers and/ or fathers, primary caregivers), we included studies meeting one of the following criteria:

1. Parents with any type of mental disorder according to the *Diagnostic and Statistical Manual of Mental Disorders* (DSM) or *International Classification of Diseases* (ICD), diagnosed using a structured assessment procedure or diagnostic technique based on earlier editions of the DSM and ICD for older studies, or based on medical record.
2. Parents with psychopathology at a clinical level (e.g., presence of significant symptoms or features), assessed by a validated (dimensional) assessment procedure. For instance, we included studies on community samples when sub samples with elevated symptom levels were reported (e.g., when a score above 13 was reported in the Beck Depression Inventory-II [BDI-II; Beck et al., 1969]).
3. Community sample with data on psychopathology at a subclinical level (e.g., self- and other-report questionnaires, interview data) in parents. For instance, psychopathology (e.g., assessed via Depression Anxiety Stress Scale-21 [DASS-21; Henry & Crawford, 2005]) in community parents was included in the statistical model on the association between emotion regulation and parenting, however no separate analysis on parents with elevated symptom levels was done.

Studies were eligible if they were published in English, Spanish, or German language. Studies were excluded if parents had a primary medical condition (e.g., cancer, HIV) or parents were diagnosed with a neurological disease due to potentially different influences on emotion regulation and/ or parenting. Furthermore, reviews, professional opinions, editorial publications, comments, or single case studies were excluded.

### Coding Procedures

**Fig. 1:**
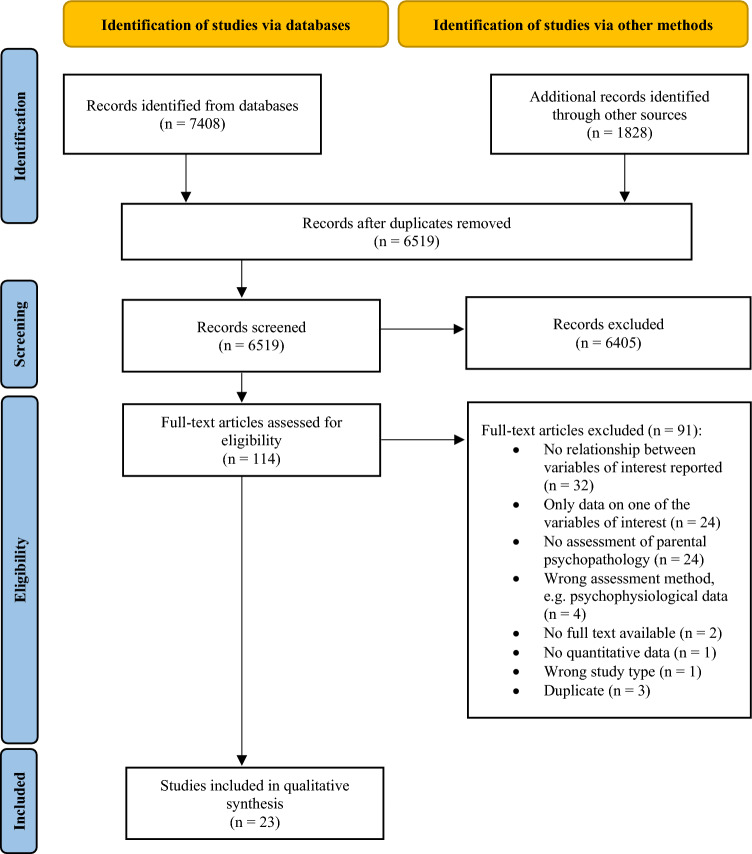
PRISMA flow diagram

All articles selected through the literature search were downloaded to a literature management program where duplicates were removed for abstract screening. In a first step, the first and the second author independently screened the titles and abstracts of all articles. Studies were eligible for subsequent full-text screening if title and/ or abstract referred to parental emotion regulation, parenting, and psychopathology. Studies being retained were organized using covidence (Veritas Health Innovation, 2019). In a second step, the first and the second author independently read full-text articles applying the above described inclusion and exclusion criteria. Reasons for the exclusion of studies following full-text review were documented (see Fig. 1). During the whole selection process, researchers were blinded concerning each other’s decisions. Disagreements between individual judgements about inclusion following title and abstract screening as well as following full-text review were resolved through discussion with the third author until a consensus was reached.

For full-text screening, almost perfect levels of agreement were reached (ĸ = 0.83; Landis & Koch, 1977). We extracted data from 23 studies (see Appendix B for extraction protocol). Among the included studies, 11 reported data on parents with mental disorders or clinical levels of psychopathology (criteria (1) or (2) from above), while 12 studies reported about parents with psychopathology at a subclinical level or provided data on psychopathology but no separate analyses on parents with elevated symptom levels (criteria (3) from above).

### Risk of Bias in Individual Studies

The first author and a research assistant independently assessed the methodological quality for each study by using adapted versions of the Joanna Briggs Institute (JBI) Critical Appraisal Checklist for analytical cross-sectional studies, cohort studies, and case–control studies (Moola et al., 2020). These tools were modified according to the use for the present systematic review and critically examined the possibility of bias in the design, conduct and analysis of the included studies. The checklists assess eight to eleven domains (e.g., the use of a valid and reliable measurement, statement regarding the identification of confounding factors and strategies to deal with them) with each to be rated as “yes,” “no,” “unclear,” or “not applicable” and contain a resulting overall appraisal. Disagreements between the raters were resolved by discussion, including the use of a third party if necessary.

The assessment of methodological quality did not reveal any severe methodological problems in the identified studies. Accordingly, all studies were included in the review (for a detailed list see Appendix C).

### Data Synthesis

Due to the heterogeneity of studies (different types of psychopathology, different measures, and designs), a narrative synthesis was conducted rather than a meta-analysis. We separate data from the clinical and subclinical populations in the results section.

## Results

### Study Characteristics

#### Geographic Region

Most of the 23 included studies (see Tables 1 and 2) were conducted in the United States of America (N = 12), six studies in Europe, and three in Australia. One study each was conducted in Japan and Rwanda.

#### Socio–economic Status

Three studies included parents with predominantly mid to high socio-economic status (see Tables 1 and 2). Samples with mixed socio-economic status were reported in seven studies. In five studies, authors indicated low income to extreme levels of poverty among the families. Eight studies did not provide detailed information on the families' socio-economic status. The studies with participants from high socio-economic status addressed depression and anxiety symptoms or diagnoses. In contrast, most studies with families from lower socio-economic status investigated effects of posttraumatic stress disorder (PTSD) and substance use disorder (SUD) on parenting.

#### Study Design

In most of the studies (*N* = 17), the study design was cross-sectional (see Tables 1 and 2). Two of the cross-sectional studies were drawn from the same study sample of a larger intervention study (Lotzin et al., 2015, 2016). Since both studies addressed different aspects of parenting, both were included in the review. Additionally, four cohort studies and two case-control studies were included.

#### Sample

Sample sizes ranged between *N* = 66 and *N* = 307 (see Tables 1 and 2). Most studies recruited parents (*N* = 21), while two focused on the recruitment of children while also collecting data on parents. The majority of the studies collected data on mothers (*N* = 14) or mother figures (*N* = 1). Five studies collected data from either mothers or fathers and two studies from primary caregivers. Only one study focused exclusively on fathers.

**Table 1:** Relationship between emotion regulation and parenting in parents with psychopathology at a clinical level

First author (year), country	Parent sample	Sample frame	Psychopathology measurement	Design	Emotion regulation measurement	Parenting aspect and measurement	Main results
Mixed diagnostic groups							
#1 Caçador (2021), Portugal	*N* = 295 mothers, *n* = 63 with clinical significant levels of anxiety and/ or depression symptoms	Urban community sample (majority with higher education, employed, higher income)	HADS^a^: Subscales Anxiety and Depression (clinical significance ≥ 11)	Cross sectional	DERS^b^-short form	Mindful Parenting (IMP^c^-infant version)	Higher levels of difficulties in emotion regulation were linked to lower levels of mindful parenting (maternal symptomatology levels did not moderate this relationship)
#2 Brake (2020), Australia	*N* = 85 mothers, *n* = 74 with mood disorder, *n* = 4 with anxiety disorders, *n* = 1 adjustment disorder, *n* = 1 hypersomnia, *n* = 5 puerperal psychosis	Inpatient sample (private health insurance, mid-high socio-economic status assumed)	ICD-10^d^ diagnosis according to medical record, EPDS^e^	Cross sectional	DERS^b^	Postnatal attachment (MPAS^f^)	Higher levels of difficulties in emotion regulation were linked to lower levels of maternal postnatal attachment
Mood disorders							
#3 Dittrich (2018), Germany	*N* = 114 mothers, *n* = 71 with major depressive disorder in remission	Outpatient sample	MINI^g^	Cross sectional	DERS^b^	Child abuse potential (CAPI^h^)	Diagnosis of maternal major depressive disorder in remission was indirectly associated with elevated abuse potential scores, mediated by severity of difficulties in emotion regulation
#4 Hien (2010), United States	*N* = 190 mothers, *n* = 40 with depressive disorder	Community sample (diverse in education)	SCID^i^ (modules for mood disorders, anxiety disorders, alcohol and psychoactive substance use disorders)	Cross sectional	NAI^j^ part A (typical emotional states and reactions to provocation)	Child abuse potential (CAPI^h^)	Anger arousal and reactivity significantly predicted child abuse potential (above and beyond diagnostic variables)
#5 Kohlhoff (2016), Australia	*N* = 84 parents (98.6% mothers) with high levels of depression symptoms (+ 1 SD)	Community sample (largely representative, majority employed full- or part-time, in occupations), families of children with conduct problems	EPDS^e^	Cross sectional	ERQ^k^	Positive and negative parenting practices (DPCIS^l^), dysfunctional discipline practices (PS^m^)	DPCIS^l^: High levels of suppression were protective against negative parenting (no effects for positive parenting); PS^m^: High levels of cognitive reappraisal were protective against hostile discipline (no effects for lax and over-reactive discipline)
#6 Lotzin (2015), Germany	*N* = 68 mothers with mood disorder	Outpatient sample (socio-economic status ranged from lower to upper middle-class)	SCID^i^(-I); BDI^n^	Cross sectional	DERS^b^	Gaze synchrony (MRSS^o^ and IRSS^p^)	Mothers with greater difficulties in emotion regulation displayed heightened mother-infant gaze synchrony (extreme attentiveness reflecting interactive dysregulation) in stressful interactions but also in general play
#7 Lotzin (2016), Germany	*N* = 68 mothers with mood disorder	Outpatient sample (socio-economic status ranged from lower to upper middle-class)	SCID^i^(-I); SCL-90-R^q^	Cross sectional	DERS^b^	Facial affect synchrony (maternal and infant facial affect rating scales)	Mothers with greater difficulties in emotion regulation displayed heightened mother-infant facial affect synchrony (reflecting interactive dysregulation)
Borderline personality disorder (BPD)							
#3 Dittrich (2018), Germany	*N* = 114 mothers, *n* = 19 with BPD	Outpatient sample	IPDE^r^	Cross sectional	DERS^b^	Child abuse potential (CAPI^h^)	Diagnosis of maternal BPD was indirectly associated with elevated abuse potential scores, mediated by severity of difficulties in emotion regulation
#8 Hiraoka (2016), United States	*N* = 133 parents (58.5% female), *n* = 15 with high levels of BPD and high risk of child abuse potential	Community sample (socio-economic status ranged from low to middle class)	MSI-BPD^s^ (high levels ≥ 7)	Case control	DERS^b^	Child abuse potential (CAPI^h^, high risk scores ≥ 166)	Difficulties in emotion regulation partially accounted for the relationship between higher BPD features and elevated child abuse potential risk
#9 Kiel (2017), United States	*N* = 99 mothers, *n* = 23 with high levels of BPD symptoms	Community sample	BEST^t^ (high levels > 30)	Cross sectional	DERS^b^	Emotion socialization (CTNES^u^)	Maternal BPD symptoms were indirectly related to increased use of punitive/ minimizing emotion socialization strategies through higher maternal difficulties in emotion regulation
Substance use disorders							
#4 Hien (2010), United States	*N* = 190 mothers, *n* = 82 with history of lifetime drug abuse or dependence	Community sample (diverse in education)	SCID^i^ (modules for mood disorders, anxiety disorders, alcohol and psychoactive substance use disorders)	Cross sectional	NAI^j^ Part A (typical emotional states and reactions to provocation)	Child sbuse potential (CAPI^h^)	Anger arousal and reactivity significantly predicted child abuse potential (above and beyond diagnostic variables)
#10 Stover (2013), United States	*N* = 86 fathers, *n* = 43 with substance abuse and intimate partner violence	Clinical and community sample (low-income families)	Screening (DSM^v^-IV criteria for substance abuse of alcohol, cocaine, or marijuana; substance use within the last 30 days); BSI^w^	Case control	DERS^b^	Positive (PRQ^x^) and negative (PRQ^x^; PARQ^y^) parenting practices	Difficulties in emotion regulation mediated the association between substance abuse and intimate partner violence group and negative parenting behaviors (but not positive parenting)
Posttraumatic stress disorder (PTSD)							
#11 Kumar (2019), United States	*N* = 78 mothers, *n* = 19 with PTSD	Community sample	Report of criterion A trauma on LEC-5^z^; CAPS-5^aa^	Cross sectional	DERS^b^	Dysfunctional discipline practices (PS^ab^)	Maternal PTSD status was indirectly related to higher maternal laxness (but not overreactivity) through greater difficulties in emotion regulation

^a^Hospital Anxiety and Depression Scale

^b^Difficulties in Emotion Regulation Scale

^c^Interpersonal Mindfulness in Parenting Scale

^d^International Classification of Diseases, Tenth Revision

^e^Edinburgh Postnatal Depression Scale

^f^Maternal Postnatal Attachment Scale

^g^Mini-International Neuropsychiatric Interview

^h^Child Abuse Potential Inventory

^i^Structured Clinical Interview for DSM

^j^Novaco Anger Inventory

^k^Emotion Regulation Questionnaire

^l^Dyadic Parent–Child-Interaction Coding System

^m^Parenting Scale

^n^Beck Depression Inventory

^o^Maternal Regulatory Scoring System

^p^Infant Regulatory Scoring System

^q^Symptom Checklist-90-Revised

^r^International Personality Disorder Examination

^s^McLean Screening Instrument for Borderline Personality Disorder

^t^Borderline Evaluation of Severity over Time

^u^Coping with Toddler’s Negative Emotions Scale

^v^Diagnostic and Statistical Manual of Mental Disorders

^w^Brief Symptom Inventory

^x^Parenting Relationship Questionnaire

^y^Parental Acceptance Rejection Questionnaire

^z^Life Events Checklist for DSM-5

^aa^Clinician-Administered PTSD Scale for DSM-5

^ab^Parenting Scale

**Table 2:** Relationship between emotion regulation and parenting in parents with psychopathology at a subclinical level

First author (year), country	Parent sample	Sample frame	Psychopathology Measurement	Design	Emotion Regulation Measurement	Parenting Aspect and Measurement	Main results
#1 Bao (2020), Japan	*N* = 300 mothers	Community sample (majority housewives)	Symptoms of anxiety (STAI^a^, clinical significance > 46) and/ or depression (CES-D^b^, clinical significance > 16)	Cross sectional	ERQ^c^	Emotion socialization (PMEPA^d^)	After controlling for parental anxiety and depression symptoms: More cognitive reappraisal was associated with greater use of supportive emotion socialization (coaching) and less unsupportive emotion socialization (dismissing), more expressive suppression was associated with less supportive emotion socialization (coaching) and more use of unsupportive emotion socialization (dismissing, non-involvement, dysfunction)
#2 Behrendt (2019), Germany	*N* = 66 mothers	Community sample (well-educated, well-situated)	Depression symptoms (BDI-II^e^)	Cohort	DERS^f^	Maternal sensitivity (EAS^g^, fourth edition); Postnatal attachment (MPAS^h^)	EAS^g^: Difficulties in emotion regulation was not related to maternal sensitivity; MPAS^h^: Difficulties in emotion regulation was related to poorer postnatal attachment
#3 Bertie (2021), Australia	*N* = 300 parents or primary caregivers	Community sample	Symptoms of anxiety and/ or depression (DASS^i^-21 subscales depression, anxiety)	Cross sectional	ERQ^c^	Emotion socialization (CCNES^j^)	Depression symptoms: More expressive suppression respectively lower cognitive reappraisal mediated the association between depression symptoms and more frequent use of non-supportive emotion socialization respectively less frequent use of supportive emotion socialization; Anxiety symptoms: No effects
#4 Casline (2020), United States	*N* = 350 parents	Community sample	Anxiety symptoms (composite score from DASS^i^-21 anxiety subscale, GAD-7^ k^, SIAS^l^, OCI-R^m^)	Cross sectional	Cognitive risk factors (DII^n^; ASI^o^; TFII^p^; PTQ^q^)	Anxiogenic parenting practices (PAKR-PR^r^)	DII^n^: Indirect association between maternal anxiety and anxiogenic parenting practices through distress intolerance (only among children with high bot not low levels of anxiety), no indirect associations among fathers; TFII^p^: Partial indirect association between parental anxiety and anxiogenic parenting practices through impulsivity; ASI^o^ and PTQ^q^: No indirect associations
#5 Doba et al. (2022), France	*N* = 72 mothers	Community sample	Symptoms of anxiety (STAI^a^-State) and/ or depression (EPDS^s^)	Cross sectional	DERS^f^	Mother-infant synchrony (verbal, motor, gaze)	Anxiety symptoms: Indirect association between maternal difficulties in emotion regulation and interactive synchrony through anxiety symptoms; Depression symptoms: No indirect associations
#6 Gurtovenko (2020), United States	*N* = 75 mothers	Community sample	Posttraumatic stress disorder symptoms (PDS^t^)	Cross sectional	Meta-Emotion Coding System applied to PMEI^u^	Emotion socialization (CCNES^j^)	Higher posttraumatic stress disorders symptoms were indirectly related to decreased supportive emotion socialization through poorer emotion regulation; No indirect effect for non-supportive emotion socialization
#7 Jensen (2021), Rwanda	*N* = 732 caregivers (92% parents), *n* = 331 with elevated internalizing symptoms, *n* = 136 with elevated posttraumatic stress disorder symptoms	Community sample (majority less than primary education, most extreme level of poverty)	Internalizing symptoms (HSCL^v^); Posttraumatic stress disorder symptoms (PCL-C^w^)	Cohort	DERS^f^	Parental acceptance and rejection (PARQ^x^)	Internalizing symptoms: Indirect effect of internalizing symptoms on decreased acceptance and increased rejection through more difficulties in emotion regulation; Posttraumatic stress disorder symptoms: Partial indirect effect of posttraumatic stress disorder symptoms on decreased acceptance through more difficulties in emotion regulation, no indirect effect for rejection
#8 Mazursky-Horowitz (2015), United States	*N* = 234 mothers	Community sample	Attention-deficit/ hyperactivity disorder symptoms T2 (CAARS^y^-S)	Cohort	DERS^f^	Emotion socialization (CCNES^j^-Adolescent Version)	Harsh responses: Difficulties in emotion regulation mediated the association between attention-deficit/ hyperactivity disorder symptoms and harsh responses; Distress and positive responses: Difficulties in emotion regulation was no mediator
#9 McCurdy (2022), United States	*N* = 80 caregivers	Community sample (majority low socio-economic status)	Anxiety symptoms (composite score from STAI^a^, BSI^z^)	Cross sectional	DERS^f^	Positive and negative parenting practices (APQ^aa^)	Difficulties in emotion regulation was no moderator of the association between anxiety symptoms and parenting
#10 Price (2021), United States	*N* = 175 mothers	Community sample (diverse in socio-economic status)	Anxiety symptoms (composite score, average of PSWQ^ab^ and SIAS^l^ means)	Cohort	DERS^f^	Emotion socialization (CTNES^ac^)	Greater maternal anxiety rather than maternal difficulties in emotion regulation predicted increased use of non-supportive emotion socialization; No relations for supportive emotion socialization
#11 Powers (2021), United States	*N* = 98 mother figures (90% biological mothers; 10% grandmothers with legal and at least 50% physical custody)	Community sample (low levels of education, predominantly low income)	Symptoms or diagnosis of depression (BDI-II^e^, score ≥ 14 presence of probable depression) and/ or posttraumatic stress disorder (CAPS^ad^) and/ or substance use (composite substance use score from all past 12-month items of AUDIT^ae^ and DAST^af^)	Cross sectional	DERS^f^	Dysfunctional discipline practices (PS^ag^), Positive parenting practices (PQ^ah^)	PS^ag^: Difficulties in emotion regulation was associated with greater overreactivity and more use of dysfunctional discipline practices in general (psychological symptoms had no effect beyond difficulties in emotion regulation); PQ^ah^: Depression symptoms were associated with lower maternal warmth above and beyond the effects of difficulties in emotion regulation
#12 Raveau (2019), United States	*N* = 96 parents	Community sample (urban sample, low in socio-economic status)	Posttraumatic stress disorder symptoms (PDS^t^)	Cross sectional	EDS^ai^	Positive parenting (Family Interaction Drawing Task coded using parent-level rating scales)	Posttraumatic stress disorder symptoms and emotion dysregulation were not related to positive parenting

^a^State-Trait Anxiety Inventory

^b^Center for Epidemiologic Studies Depression Scale

^c^Emotion Regulation Questionnaire

^d^Parental Meta-Emotion Philosophy about Anger Questionnaire

^e^Beck Depression Inventory-Revised

^f^Difficulties in Emotion Regulation Scale

^g^Emotional Availability Scales

^h^Maternal Postnatal Attachment Scale

^i^Depression Anxiety Stress Scale

^j^Coping with Children’s Negative Emotions Scale

^k^Generalized Anxiety Disorder 7-item scale

^l^Social Interaction Anxiety Scale

^m^Obsessive Compulsive Inventory Revised

^n^Distress Intolerance Index (6-item version with highest-loading items)

^o^Anxiety Sensitivity Index 3 (9-item version with the three highest-loading items from each subscale)

^p^Three Factor Impulsivity Index (Feelings Trigger Action subscale)

^q^Perseverative Thinking Questionnaire (5-item version with highest-loading items)

^r^Parenting Anxious Kids Rating Scale—Parent Report

^s^Edinburgh Postnatal Depression Scale

^t^Posttraumatic Stress Diagnostic Scale

^u^Parent Meta-Emotion Interview

^v^Hopkin’s Symptoms Check List

^w^Posttraumatic Stress Disorder Checklist-Civilian Version

^x^Parental Acceptance-Rejection Questionnaire

^y^Conner’s Adult ADHD Rating Scale

^z^Brief Symptom Inventory

^aa^Alabama Parenting Questionnaire

^ab^Penn State Worry Questionnaire

^ac^Coping with Toddler’s Negative Emotions Scale

^ad^Clinician-Administered PTSD Scale for DSM-5

^ae^Alcohol Use Disorder Identification Test

^af^Drug Abuse Screening Test

^ag^Parenting Questionnaire

^ah^Parenting Scale

^ai^Emotional Dysregulation Scale

### What Types of Psychopathology were Investigated?

Among the included studies, nine examined different types of psychopathology, partly reporting results separately (*N* = 6) or in aggregated form (*N* = 3; see Tables 1 and 2). Though more than one diagnostic group was investigated, none of those studies made direct group comparisons. In most cases, comparisons were made with healthy control groups.

Studies on parents with mental disorders or clinical levels of psychopathology (*N* = 11; see Table 1) mostly investigated mood disorders (*N* = 5), three were on BPD, two on SUD, and one on PTSD or respective psychopathology at a clinical level.

Studies assessing psychopathology at a subclinical level in parents (*N* = 12; see Table 2) mostly focused on anxiety symptoms (*N* = 5). Four studies each focused on PTSD symptoms or depressive symptoms. One study each addressed symptoms of attention deficit and hyperactivity disorder (ADHD), internalizing symptoms and substance use.

### How was Emotion Regulation Operationalized in the Context of Parenting?

As expected, we observed heterogeneity regarding concepts and operationalizations of emotion regulation. We classified the operationalizations of emotion regulation encountered in the identified studies according to three broader concepts: More general *abilities in or difficulties in emotion regulation*, *modulation of emotion activation*, and *experience and expression of emotions* (see Table 3).

**Table 3:** Concepts and measurements of emotion regulation used in the included studies

Concept	Measurements
Abilities in or difficulties in emotion regulation	Difficulties in Emotion Regulation Scale (DERS; Gratz & Roemer, 2004)
	Emotional Dysregulation Scale (EDS; Westen et al., 1997)
Modulation of emotion activation	Emotion Regulation Questionnaire (ERQ; Gross & John, 2003)
	Perseverative Thinking Questionnaire (PTQ; Ehring et al., 2011)
Experience and expression of emotions	Parent Meta-Emotion Interview (PMEI; Katz & Gottman, 1986) and Meta-Emotion Coding System (Katz et al., 1994)
	Novaco Anger Inventory (NAI; Novaco, 1994, 2003)
	Distress Intolerance Index (DII; McHugh & Otto, 2011, 2012)
	Anxiety Sensitivity Index 3 (ASI-3; Taylor et al., 2007)
	Feelings Trigger Action subscale of the Three Factor Impulsivity Index (TFII; Carver et al., 2011; Johnson et al., 2013)

Most studies assessed *general difficulties in emotion regulation* (*N* = 17) via self-report questionnaires. The Difficulties in Emotion Regulation Scale (DERS; Gratz & Roemer, 2004) was used in 16 studies and provides a multidimensional conceptualization of emotion regulation ranging from the awareness and acceptance of emotions to the ability to engage in goal-directed behavior (Gratz & Roemer, 2004). A similar conceptualization can be found in the Emotional Dysregulation Scale (EDS) used in one study. The EDS is a self-report questionnaire based on the clinician-rated Affect Regulation and Experience Q-sort Questionnaire (Westen et al., 1997).

The *modulation of emotion activation* through the self-reported use of the strategies cognitive reappraisal and expressive suppression was assessed in three studies using the Emotion Regulation Questionnaire (ERQ; Gross & John, 2003).

Two studies used operationalizations with a focus on the *experience and the expression of emotions*: The Parent Meta-Emotion Interview (PMEI; Katz & Gottman, 1986) and the Meta-Emotion Coding System (Katz et al., 1994) broadly capture the experiential dimension of emotion regulation. It allows to assess both, self-rated and observer-rated experiences with and attitudes toward the emotions of fear and anger and their regulation (Katz & Gottman, 1986; Katz et al., 1994). In another study, the expression of anger was assessed by using the Novaco Anger Inventory (NAI; Novaco, 1994, 2003). This questionnaire assesses anger arousal and reactivity by having participants indicate how typical emotional states and responses to provocation are for them (Novaco, 1994, 2003).

Finally, in one study, four parental cognitive risk factors related to the *experience, expression and modulation of emotions* were examined using respective self-report questionnaires: An abbreviated version of the Distress Intolerance Index (DII; McHugh & Otto, 2011, 2012) measuring the parent’s felt tolerance of aversive emotions and physical states; an abbreviated version of the Anxiety Sensitivity Index 3 (ASI-3; Taylor et al., 2007) capturing the parent’s conviction that experiencing fear has negative consequences; the Feelings Trigger Action subscale of the Three Factor Impulsivity Index (TFII; Carver et al., 2011; S. L. Johnson et al., 2013) measuring the parent’s emotion-related impulsivity; and an abbreviated version of the Perseverative Thinking Questionnaire (PTQ; Ehring et al., 2011) assessing repetitive negative thinking among parents.

### Which Aspects of Parenting were Investigated?

As expected, we also observed substantial heterogeneity regarding concepts and operationalizations of parental behavior. We identified four main concepts in the field of parenting research: *Parenting with positive valence*, *parenting with negative valence*, *parental emotion socialization*, and *anxiogenic parenting practices* (see Table 4).

**Table 4:** Global concepts, related aspects of parenting and operationalizations used in the included studies

*Aspects of parenting with positive valence*
Using self-report measures and observational coding, several aspects of parenting with positive valence were assessed. Related dimensions included: Parental warmth, sensitivity, acceptance, (postnatal) attachment, involvement, cohesiveness, (emotional) support, (un)labeled praise, and mindful parenting. • Self-report measures: Alabama Parenting Questionnaire (Frick, 1991); Parenting Questionnaire (McCabe et al., 1999); Parental Acceptance-Rejection Questionnaire (Rohner et al., 2005); Parenting Relationship Questionnaire (Kamphaus & Reynolds, 2006); Maternal Postnatal Attachment Scale (Condon & Corkindale, 1998); Interpersonal Mindfulness in Parenting Scale (- Infant version; Caiado et al., 2020; Duncan, 2007; Moreira & Canavarro, 2017) • Observational coding systems: Dyadic Parent–Child Interaction Coding System (Eyberg et al., 2010); The Family Interaction Drawing Task (Cox et al., 1998; Lindahl & Malik, 2001; McHale & Fivaz-Depeursinge, 1999); Emotional Availability Scales (Biringen et al., 2014)
*Aspects of parenting with negative valence*
Similarly, several aspects of parenting with negative valence were assessed using self-report measures and observational coding. Related dimensions included: Dysfunctional discipline practices (laxness, over-reactivity, and hostility), corporal punishment, negative talk, rejection, demandingness, and relational frustration. Furthermore, a heightened synchrony in the mother-infant interaction (motor, gaze, facial affect, verbal) was interpreted as indicators of less favorable parenting behavior. Finally, child abuse potential was assessed as a severe form of negative parenting. • Self-report measures: Alabama Parenting Questionnaire (Frick, 1991); Parenting Questionnaire (McCabe et al., 1999); Parenting Scale (Arnold et al., 1993); Parental Acceptance-Rejection Questionnaire (Rohner et al., 2005); Parenting Relationship Questionnaire (Kamphaus & Reynolds, 2006); Child Abuse Potential Inventory (Milner et al., 1986) • Observational coding systems: Dyadic Parent–Child Interaction Coding System (Eyberg et al., 2010); Parent–child gaze/facial affect synchrony (Lotzin et al., 2015, 2016); Coding of mother-infant interaction dynamics (verbal, motor, and gaze; Doba et al., 2022)
*Parental emotion socialization*
This concept comprises parental supportive and non-supportive responses in managing negative emotions in children. Using age-specific versions of self-report questionnaires, parents judge typical reactions: Coping with Toddler’s Negative Emotions Scale (Spinrad et al., 2007); Coping with Children’s Negative Emotions Scale (- Adolescent Version; Fabes et al., 1990); Parental Meta-Emotion Philosophy about Anger Questionnaire (- Japanese version; Bao & Kato, 2020).
*Anxiogenic parenting practices*
One study specifically investigating the role of transdiagnostic risk factors to explain the link between parent anxiety and anxiety-specific parenting behaviors used the Parenting Anxious Kids Rating Scale (- Parent Report; Flessner et al., 2017).

The concepts of *parenting with positive and negative valence* encompass a wide range of aspects evident in behavioral, interactional, and attitudinal dimensions of parenting that can be evaluated as favorable or unfavorable. Most of the included studies investigated parenting with negative (*N* = 12) or positive valence (*N* = 9), with five of those studies considering both aspects. Parenting with positive and negative valence was either investigated using self-report measures or observational coding. While self-report was used among parents with offspring comprising a wide age range from 0 months to 15 years, observational coding was predominantly applied in samples with younger children/ infants between the age of 6 and 32 months and varied to the extent that there were specifications to the task (e.g., free play or drawing task).

The concept* parental emotion socialization* refers to parental responses in managing negative emotions in their children and was investigated in six studies. It was also examined across a wide age range from 12 months to 14 years.

In parents with anxiety symptoms (*N* = 1), the use of *anxiogenic parenting practices* in the interaction with their relatively older children (mean age 10 years) was investigated in order to study intergeneration transmission of anxiety. This concept focuses on anxiety-specific parenting behaviors like over-involvement that are assumed to enhance anxiety in children (Casline et al., 2020).

### How was Emotion Regulation in Parents with Psychopathology at a Clinical Level Related to Different Aspects of Parenting?

Results from studies including parents with psychopathology at a clinical level (*N* = 11) are summarized in Table 1.

#### Mental Disorders in General

In samples of mothers with different mental disorders, difficulties in emotion regulation were associated with decreased positive parenting, more specifically with reduced attachment quality (Brake et al., 2020) and decreased mindful parenting (Caçador & Moreira, 2021). Interestingly, maternal psychopathology levels did not moderate the latter association. Also, among mothers with subclinical mental distress, difficulties in emotion regulation were associated with decreased mindful parenting (Caçador & Moreira, 2021).

#### Mood Disorders

Among parents with mood disorders, there was a clear link between deficits in emotion regulation and negative (but not positive) parenting. Furthermore, specific emotion regulation strategies appeared to provide a buffer against negative parenting. Lotzin and colleagues observed that difficulties in emotion regulation were related to heightened mother–infant gaze synchrony and facial affect synchrony. Both are seen as indicators of interactive dysregulation reflecting extreme attentiveness (Lotzin et al., 2015, 2016). Furthermore, two studies reported general emotion regulation difficulties (Dittrich et al., 2018) and difficulties with the expression of emotions in terms of higher anger arousal and reactivity (Hien et al., 2010) to be associated with elevated levels of child abuse potential. The observed relationships were independent of contextual (stressful situation; Lotzin et al., 2015, 2016), demographic (marital status), and diagnostic (comorbidities; Hien et al., 2010) variables. Kohlhoff et al. (2016) focused on specific emotion regulation strategies and found higher levels of cognitive reappraisal to be associated with less use of negative parenting (e.g., hostile discipline), but not with positive parenting. Similarly, higher levels of suppression were associated with less negative (but not with positive) parenting behavior (Kohlhoff et al., 2016).

#### Borderline Personality Disorder

Among parents with BPD, deficits in emotion regulation were robustly linked to negative parenting and non-supportive emotion socialization. Hiraoka et al. (2016) found emotion regulation difficulties to partly account for the relationship between elevated BPD features and increased child abuse potential. Similarly, Dittrich et al. (2018) showed that severity of emotion regulation difficulties mediated the association between the diagnosis of maternal BPD and increased child abuse potential. Kiel et al. (2017) studied maternal emotion socialization and found BPD symptoms in mothers to be indirectly associated with an increased use of punitive/ minimizing emotion socialization strategies via maternal difficulties in emotion regulation.

#### Substance Use Disorders

Among parents with SUD, emotion regulation difficulties were linked to negative parenting. In accordance with the findings on parents with mood disorders, difficulties with the expression of emotions in terms of higher anger arousal and reactivity predicted elevated levels of child abuse potential, indicating no diagnostic specificity (Hien et al., 2010). Stover et al. (2013) compared men with substance abuse and intimate partner violence to community control fathers. They found a significant association between clinical group membership and negative parenting behaviors (e.g., rejection, hostility), mediated by difficulties in emotion regulation. However, in the context of positive parenting and co-parenting, difficulties in emotion regulation did not serve as a mediator (Stover et al., 2013).

#### Posttraumatic Stress Disorder

Kumar et al. (2019) observed an indirect effect of maternal PTSD on some aspects of negative parenting behaviors (lax but not overreactive) through elevated difficulties in emotion regulation.

### How was Emotion Regulation in Parents with Psychopathology at a Subclinical Level Related to Different Aspects of Parenting?

In 12 studies, the association between emotion regulation and parenting was explored among parents with psychopathology at a subclinical level (see Table 2).

#### Elevated Level of Psychopathology

In samples of mothers with mixed psychopathological symptoms, there is evidence that both emotion regulation capacities and depressive symptoms were linked to more negative parenting. Furthermore, reappraisal strategies showed beneficial effects, and expressive suppression detrimental effects on parental emotion socialization when controlling for psychopathology. Jensen et al. (2021) examined the longitudinal effects of several psycho-social risk factors on parenting behavior in a rural community sample of caregivers with high levels of poverty. They found that higher internalizing symptoms and subsequent greater difficulties in emotion regulation predicted less accepting and more rejecting parenting (Jensen et al., 2021). Among mothers in a low socio-economic community sample, difficulties in emotion regulation predicted overall dysfunctional parenting behavior and overreactivity, while substance use and trauma-associated and depressive symptoms had no significant contribution (Powers et al., 2021). Bao and Kato (2020) studied the association between specific emotion regulation strategies and emotion socialization in mothers from a community sample controlling for symptoms of anxiety and depression. They found reappraisal strategies to be related to more supportive emotion socialization (coaching) and fewer unsupportive emotion socialization (dismissing). Expressive suppression, in turn, was related to fewer supportive emotion socialization (coaching) and more unsupportive emotion socialization (dismissing, non-involvement, dysfunction; Bao & Kato, 2020).

#### Anxiety Symptoms

In samples in which parental anxiety symptoms were examined, neither emotion regulation difficulties nor specific emotion regulation strategies were associated with parenting or parental emotion socialization. However, for interpersonal difficulties in direction of hypervigilant parenting (e.g., anxiogenic parenting behavior or mother–infant interactive synchrony), emotion dysregulation seemed to be of higher relevance. In a community-based sample, Price and Kiel (2021) found maternal anxiety rather than difficulties in emotion regulation to be predictive of non-supportive emotion socialization strategies. For supportive emotion socialization, no significant associations were observed. Similarly, McCurdy et al. (2022) observed a negative effect of parental anxiety symptoms on their parenting. However, no indirect association between anxiety symptoms and positive or negative parenting via emotion regulation was found. With a focus on specific emotion regulation strategies, Bertie et al. (2021) did not observe a mediating role of applying expressive suppression and cognitive reappraisal regarding the association between anxiety symptoms and emotion socialization strategies among community parents. In contrast to Price and Kiel (2021) and McCurdy et al. (2022), parental anxiety was not associated with non-supportive emotion socialization (Bertie et al., 2021). Casline et al. (2020) addressed the individual contribution of different transdiagnostic cognitive risk factors in community parents for anxiogenic parenting behaviors and child anxiety. They reported that impulsivity rather than anxiety sensitivity or perseverative thinking partially explained the association between parental anxiety and anxiogenic parenting behavior. Only among children with high (but not low) anxiety levels, a positive relationship between maternal anxiety and distress intolerance was found, which in turn predicted greater anxiogenic parenting behavior (Casline et al., 2020). In line with this finding, Doba et al. (2022) observed that maternal anxiety symptoms mediated the relationship between greater difficulties in emotion regulation and higher synchrony in the mother–infant interaction (in gaze, verbal, and motor behavior), reflecting hypervigilant maternal behavior.

#### Symptoms of Depression

In the study of depressive symptoms in parents, difficulties in emotion regulation and depressive symptoms had differential effects on diminished positive parenting. Furthermore, certain emotion regulation strategies appeared to be particularly important for emotion socialization. In a community sample of first-time mothers, depressive symptoms were associated with difficulties in emotion regulation; However, they predicted different aspects of parenting: While difficulties in emotion regulation led to poorer postnatal bonding, depressive symptoms were associated with less maternal sensitivity (Behrendt et al., 2019). Accordingly, depressive symptoms rather than difficulties in emotion regulation were predictive of lower maternal warmth among mothers in a low socioeconomic community sample (Powers et al., 2021). Doba et al. (2022) reported an association between maternal difficulties in emotion regulation and depressive symptoms, although no effects on synchrony in mother–child interaction were found. Looking at specific emotion regulation strategies, Bertie et al. (2021) observed an indirect effect between symptoms of depression and both, supportive and non-supportive emotion socialization strategies, via expressive suppression and cognitive reappraisal. Depression symptoms were associated with increased expressive suppression and decreased cognitive reappraisal, subsequently leading to more non-supportive and less supportive emotion socialization (Bertie et al., 2021).

#### Posttraumatic Stress Disorder Symptoms

Among parents with PTSD symptoms, difficulties in emotion regulation seemed to reduce positive parenting and supportive emotion socialization. However, findings were inconsistent. In their aforementioned study on longitudinal effects of several psycho-social risk factors, Jensen et al. (2021) reported that higher PTSD symptoms and subsequent greater difficulties in emotion regulation predicted less accepting parenting. Gurtovenko and Katz (2020) studied mothers who experienced intimate partner violence and found PTSD symptoms to be indirectly related to decreased use of supportive emotion socialization via poorer emotion regulation. No such effect was observed for rejecting parenting (Jensen et al., 2021) or non-supportive emotion socialization (Gurtovenko & Katz, 2020). In contrast to the previous findings, Raveau (2019) observed no associations between PTSD symptoms, difficulties in emotion regulation, and positive parenting in an urban community sample of parents with high levels of poverty.

#### Attention Deficit and Hyperactivity Disorder Symptoms

Considering ADHD symptoms, difficulties in emotion regulation appeared to influence individual aspects of maternal emotion socialization. Using data from a prospective longitudinal study, Mazursky-Horowitz et al. (2015) showed that ADHD symptoms of mothers in a community sample were indirectly related to the use of harsh emotion socialization strategies through difficulties in emotion regulation. However, no such relationship was found for distress responses or supportive emotion socialization.

## Discussion

Our primary goal was to summarize research on how emotion regulation in parents with mental disorders is related to their parenting. Although emotion dysregulation is assumed to be a transdiagnostic risk factor for psychopathology (Aldao et al., 2010), its specific association with parenting across mental disorders is not systematically considered in past reviews. With the aim to provide effective intervention options and thus interrupt the intergenerational transmission of difficulties in emotion regulation, it is important to accumulate knowledge about existing difficulties and relevant conditions. Therefore, we systematically reviewed research on the interrelationship between emotion regulation and parenting in the context of parental mental health conditions.

To summarize our findings, difficulties in emotion regulation among parents with psychopathology at a clinical and subclinical level were associated with several aspects of unfavorable parenting outcomes, with more robust findings for parenting with negative than positive valence. Moreover, differences were observed dependent on psychopathology investigated. In some diagnostic groups (e.g., depressive disorders, BPD, SUD), emotion regulation in parents was relevant for parenting capacities beyond the contribution of the specific psychopathology. In contrast, associations between emotion dysregulation and parenting have not been observed among parents with anxiety disorders. For the use of specific emotion regulation strategies, the association with parenting was less consistent.

When taking a closer look at the studies of parents with psychopathology at a clinical level, our findings show that difficulties in emotion regulation were robustly associated with several aspects of negative parenting (e.g., interactive dysregulation, lax parenting), regardless of the type of diagnosed mental disorder. Also, child abuse potential—investigated in parents with depressive disorder, BPD and SUD—and parental non-supportive reactions toward their children’s negative emotions was consistently affected by parents’ difficulties in emotion regulation. An exception here were mothers with a PTSD, for whom only some aspects of negative parenting behavior (laxness but not overreactivity) were influenced by their difficulties in emotion regulation. For positive parenting (e.g., postnatal attachment, mindful parenting), the association with emotion regulation was slightly less consistent, but at the same time studied less frequently. Most research on parents with mental disorders has focused on negative parenting (*N* = 10), and only a few studies (*N* = 3) have examined positive parenting.

When including the studies on parents with psychopathology at a subclinical level, we observed a slightly greater balance between studies with a focus on negative and positive parenting outcomes. Although we generally found similar results, findings in this group were less consistent and we observed differential effects dependent on the investigated mental health problem.

Overall, our findings align with those of Zimmer-Gembeck et al. (2022), who recently published a meta-analysis on the association between emotion regulation and parenting in non-clinical samples. They reported an association between emotion dysregulation, respectively fewer strategy use and unfavorable parenting behaviors. More specifically, higher effect sizes and consistency in research findings were noted when negative parenting behavior but not supportive parenting was investigated. The authors suggested a slight specificity of the effect of parental emotion regulation on negative parenting (Zimmer-Gembeck et al., 2022). In line with our observation, they identified more studies with a focus on negative than on positive ﻿parenting (Zimmer-Gembeck et al., 2022); However, the disparity was not as great as in the summary of studies on parents with mental disorders. Accordingly, emotion dysregulation seems to be central for parenting difficulties in general and parental psychopathology appears to be of additional relevance.

In this regard, we made another interesting observation: While positive and negative parenting were equally affected by emotional dysregulation in mixed diagnostic groups, positive parenting was less affected at the individual level of some mental problems (e.g., depressive disorders, SUD, ADHD symptoms). As research suggests, the presence of a parental mental health condition is linked to a potential decline in the quality of parent–child interaction, as observed in the included articles and previous studies (e.g., Johnson et al., 2006). Moreover, there might be additional factors, aside from emotion regulation, that mitigate the ability of parents with specific mental health conditions to demonstrate positive parenting. As per the widely used and empirically supported process model of parenting proposed by Belsky (1984) and the more recent update by Taraban and Shaw (2018), various factors jointly play a role in influencing parenting behavior. These factors include parental characteristics (such as personality and developmental history), child characteristics (such as negative emotionality), and the family and social environment (such as marital quality, social support, and culture; Belsky, 1984; Taraban & Shaw, 2018). Regarding parental characteristics, one aspect might be parental mentalization, as the ability to enter the child’s subjective world helps parents to show sensitive and supportive parenting behaviors (Camoirano, 2017). Also, disorder-specific aspects in cognition, attribution, and in the interactional behavior might explain the decrease in positive parenting. Regarding the family and social environment, social support might contribute to the observations as it plays a central role in facilitating positive parenting behaviors when parents suffer from mental disorders (Seeger et al., 2022). Some of the included studies give indications of other possible relevant variables: Brake et al. (2020) performed additional analysis including further variables and showed that women with insecure attachment and related emotion regulation difficulties had an increased risk of postnatal depression and subsequent impaired postnatal attachment quality to their children. Similarly, Stover et al. (2013) pointed out that paternal avoidant attachment contributes to their parenting in addition to emotional dysregulation. Dittrich et al. (2018) and Jensen et al. (2021) emphasize the role of early life maltreatment and experienced trauma for psychopathology, emotion regulation difficulties, and subsequent parenting difficulties. Finally, Jensen et al. (2021) and Raveau (2019) pointed to an additional contribution of caregiver hardship and demographic risk factors (e.g., low income and education) for the prediction of parenting. Future studies are needed to test the theoretical assumptions and empirical considerations described above. Moreover, a complex interplay between emotion regulation and mentalization (Schultheis et al., 2019) as well as social support and other determinants in the context of parenting should be considered.

For the role of specific emotion regulation strategies, conclusions are less certain. We found inconsistent results, while we also observed a small number of studies (*N* = 4) on this aspect of emotion regulation. Only for depressive disorders, strategy use (suppression, reappraisal) seemed to buffer against the emergence of negative parenting behavior (e.g., hostile discipline, non-supportive emotion socialization). This is in line with the observations made by Zimmer-Gembeck et al. (2022) who reported inconsistent findings and smaller effect sizes for strategy use as compared with general abilities in emotion regulation. In the study of strategy use, methodological aspects might be of further importance. In the included studies, the use of emotion regulation strategies was assessed via self-report questionnaires without a focus on the appropriateness of the strategy in the respective context or for the investigated parenting outcome. While specific emotion regulation strategies have been considered as variably adaptive in the past, the flexibility of strategy use dependent on contextual and individual factors has been increasingly emphasized in recent years (e.g., Aldao et al., 2015; Gross, 2015). This indicates that focusing purely on strategies without considering the context or the success of implementation may not be particularly informative (Lincoln et al., 2022). Especially when interacting with children, suppression might help to reduce the intensity of negative emotions and prevent dysfunctional reactions toward the child (Zimmer-Gembeck et al., 2022). Investigating strategy use directly in the interaction with the child might help to study the flexibility and appropriateness in future studies.

### Psychopathology-specific Findings

For parents with depressive disorders or symptoms who represented the most frequently studied group, there was clear evidence that difficulties in emotion regulation increase the likelihood of negative parenting in the mother–child interaction (e.g., facial affect and gaze synchrony), and show an association to heightened child abuse potential and to fewer postnatal attachment to their children. Furthermore, a greater use of strategies to modulate emotional activation (e.g., suppression, cognitive reappraisal) seems to buffer against negative parenting (e.g., dysfunctional discipline practices) while positive parenting seems to be unaffected. Interestingly, in contrast to our findings, Zimmer-Gembeck et al. (2022) only observed a contribution of suppression but not of reappraisal in non-clinical samples. Hence, for parents with depressive disorders, both strategies might be relevant to decrease the likelihood of negative parenting. This supports the idea that parents with depression should be supported in their overall emotion regulation abilities and in expanding their repertoire of strategies to reduce negative parenting (e.g., Behrendt et al., 2019). In addition to these emotion regulation-related associations, depressive symptomatology itself appeared to have detrimental effects on positive parenting (e.g., maternal sensitivity and warmth), suggesting an additional pathway in this regard.

For some diagnostic categories, emotion dysregulation was only associated with negative but not with positive aspects of parenting. Among parents with SUD, difficulties in emotion regulation increased the likelihood of negative parenting and child abuse potential whereas they did not interfere with parents’ positive parenting. Only one study addressed parental ADHD symptoms and found emotion dysregulation to predict unsupportive (but not supportive) emotion socialization. Among parents with BPD, parental emotion dysregulation was robustly associated with the use of unfavorable emotion socialization strategies and heightened child abuse potential. According to our search, positive aspects of parenting have not been studied in this group so far and should therefore be examined in future studies. These findings pronounce the importance to promote emotion regulation abilities in the mentioned diagnostic groups. Furthermore, pathways to promote positive parenting should be identified.

Among parents with PTSD symptoms, findings were inconsistent with some studies reporting an association between emotion regulation and parenting and others not. Interestingly, in two studies, parental PTSD symptoms and associated emotion regulation difficulties decreased the likelihood for positive parenting (e.g., acceptance, supportive emotion socialization) and led to an elevated use of lax (but not overreactive) parenting behaviors. It seems that PTSD symptoms and related difficulties in emotion regulation are associated with more permissive and overly lenient parenting rather than with elevated unfavorable active behaviors. Due to a limited number of studies and heterogenic operationalizations of parenting, future studies addressing these observations are needed.

For parents with anxiety disorder or symptoms, the psychopathology itself seems to explain unfavorable parenting behavior (e.g., unsupportive emotion socialization, anxiogenic parenting behaviors, hypervigilant parenting). Emotion regulation in general or specific strategies were not associated with negative parenting behavior in this group. Here, impulsivity and distress intolerance seem to have a stronger impact on dysfunctional parenting (Casline et al., 2020). Interestingly, hypervigilant maternal behavior was promoted by maternal difficulties in emotion regulation and subsequent increased anxiety symptoms. In contrast, no such effect was observed when depressive symptoms were considered, suggesting a differential effect in favor of anxiety symptoms on hypervigilant parenting behaviors. Due to a limited number of studies on this association, further research is needed.

### Limitations

With respect to the qualitative synthesis of the studies reviewed, we identified four major limitations that limit the generalizability of the results and should therefore be considered in the design of future research: (1) Predominantly mothers studied; (2) Lack of group comparisons; (3) Heterogeneity of variables of interest and (4) Consideration of other factors.

First, as most of the studies addressed mothers only, we can only draw conclusions for mothers and not for fathers or parents in general. Although the association between emotion regulation and parenting has been observed across several mental health problems studied in the included articles, most studies refer to mothers. Future studies should therefore also integrate fathers and other primary caregivers.

Second, most studies, with the exception of a few, focused on one diagnostic group. When comorbidities were considered or group comparisons were made, no diagnostic specificity was found regarding the presence of a relationship between emotion regulation and parenting. Conclusions regarding disorder specificity in terms of descriptively observed differences between psychopathologies on some aspects of parenting behavior are limited due to a lack of comparisons between diagnostic groups in the included studies. Nevertheless, the results summarized here can provide a basis for planning future studies. Future research should allow for group comparisons to examine whether the observed differences between psychopathologies reveal disorder-specific aspects in parenting behaviors and consequently provide appropriate interventions. In addition, considerable heterogeneity was found in the number of studies of the individual mental health problems of parents. While mostly depressive symptoms or disorders were studied, for some diagnostic groups (e.g., ADHD symptoms), only one article was identified limiting generalizability of research findings.

Third, we also observed a considerable heterogeneity regarding the operationalization of emotion regulation and parenting. Emotion regulation was predominantly assessed using self-report measures on general difficulties in emotion regulation, which makes the conclusions here more certain. In contrast, studies focusing on the experience and expression of emotions as well as on the self-reported modulation of emotional activation by the use of specific strategies (e.g., suppression, cognitive reappraisal) were underrepresented. Similarly, the global concepts of parenting with positive and negative valence were not equally represented with a balance in direction of negative aspects and measured by a wide range of indicators (e.g., parental acceptance, child abuse potential). Since positive parenting behavior is equally important for healthy child development, it should likewise be examined in future studies. Both within and between groups of disorders, comparison of the findings is limited due to the heterogeneity of the variables studied. Nonetheless, the results summarized here can be used as a starting point to plan future studies. As mentioned before, most studies relied on self-report in assessing emotion regulation and parenting behaviors, so shared variance in methods and socially desirable responses can be assumed. Although this generally allows a better comparability, future studies should use multi-method designs and investigate dynamic interactions between child and parental variables. Moreover, the association between emotion regulation and parenting was predominantly examined cross-sectionally. Longitudinal designs are warranted in order to draw causal conclusions on the role of emotion regulation.

Finally, future studies should examine other variables associated with emotion regulation and parenting (e.g., socioeconomic resources, mentalizing, child characteristics) to distinguish their individual contributions. In our literature synthesis, we observed some disparities in terms of psychopathological symptoms and family background, although a direct comparison was difficult because of study heterogeneity. While studies with parents with PTSD or SUD were mainly conducted in contexts with greater poverty, parents with depression or anxiety symptoms were predominantly studied in contexts of higher socio-economic backgrounds. Given the general evidence of the impact of adverse socio-contextual factors on unfavorable parenting (Fang et al., 2021; Marsh et al., 2020), results on the observed association between emotion regulation and parenting in both diagnostic groups should be interpreted with caution, as potentially confounding socio-contextual factors cannot be excluded. For example, one might expect social resources to protect against negative outcomes by providing a retreat to other spaces and childcare through babysitters. This might also allow for considering other emotion regulation strategies. Therefore, future studies should take socio-contextual factors into account in order to assess its influence on parenting. Interestingly, and in contrast to our findings, Zimmer-Gembeck et al. (2022) observed that in studies investigating high-risk participants (e.g., trauma or intimate partner violence in family context), no significant association between emotion regulation and parenting was observed.

### Implications

In summary, challenges with parental emotion regulation and the impact of mental health conditions can influence parenting resources and are associated with less favorable parenting outcomes. Clinicians should therefore not only provide interventions to improve psychopathological symptoms but also to promote emotion regulation, especially when patients are parents. Evidence-based treatment for BPD (dialectic behavioral therapy; Linehan, 1993) emphasizes the role of emotion regulation in the etiology of BPD and focus on the improvement of emotion regulation in therapy. In the context of parenting in individuals with BPD, interventions with a central focus on supporting parental emotion regulation have been developed in the last years (Rosenbach et al., 2020). For depressive disorders, in addition to the reduction of depressive symptoms, interventions should also consider promoting emotion regulation and the use of strategies. As findings on positive parenting were inconsistent, other variables than emotion regulation (e.g., mentalization, social resources) should be investigated in future studies in order to understand the contributing factors and provide appropriate interventions.

### Supplementary Information

Below is the link to the electronic supplementary material (Appendices A, B, and C).

Supplementary file1 (DOCX 23 KB)
