# Supplementary material for: Emotion Regulation, Parenting, and Psychopathology: A Systematic Review
Source: Clin Child Fam Psychol Rev. 2023 Sep 13;27(1):1–22. doi: 10.1007/s10567-023-00452-5 (PMC10920465; doi:10.1007/s10567-023-00452-5)
Supplement: Supplementary file 1 — Supplementary file1 (DOCX 23 KB) [file 10567_2023_452_MOESM1_ESM.docx]

Appendices:

**Appendix A: Search strategy for each database**

Medline (PubMed)

| Number | Query |
| --- | --- |
| #1 | ("parents/psychology"[MH]) OR ("caregivers/psychology"[MH]) OR (parent*[TIAB]) OR (mother*[TIAB]) OR (father*[TIAB]) OR (caregiver*[TIAB]) |
| #2 | ("emotional regulation"[MAJR]) OR ("emotion* regulat*"[TIAB]) OR ("emotion* dysregulat*"[TIAB]) OR ("affect* regulat*"[TIAB]) OR ("affect* dysregulat*"[TIAB]) |
| #3 | ("parenting/psychology"[MAJR]) OR ("maternal behavior/psychology"[MH]) OR ("paternal behavior/psychology"[MH]) OR ("parent-child relations"[MH]) OR ("child abuse/psychology"[MH]) OR (parenting[TIAB]) OR ("parent* behavi*"[TIAB]) OR ("maternal behavi*"[TIAB]) OR ("paternal behavi*"[TIAB]) OR ("parent* discipline practice*"[TIAB]) OR ("parent* negativity"[TIAB]) OR ("maternal negativity"[TIAB]) OR ("paternal negativity"[TIAB]) OR ("parent* warmth"[TIAB]) OR ("maternal warmth"[TIAB]) OR ("paternal warmth"[TIAB]) OR ("parent* control"[TIAB]) OR ("maternal control"[TIAB]) OR ("paternal control"[TIAB]) OR ("parent* hostility"[TIAB]) OR ("maternal hostility"[TIAB]) OR ("paternal hostility"[TIAB]) OR ("parent* sensitivity"[TIAB]) OR ("maternal sensitivity"[TIAB]) OR ("paternal sensitivity"[TIAB]) OR ("emotion* socialization"[TIAB]) OR ("emotion* socialisation"[TIAB]) OR ("child abus*"[TIAB]) OR ("child neglect*"[TIAB]) OR ("child maltreat*"[TIAB]) OR (((parent[TIAB]) OR (mother[TIAB]) OR (father[TIAB])) AND ((child[TIAB]) OR (infant[TIAB]) OR (baby[TIAB]) OR (toddler[TIAB]) OR (adolescent[TIAB]) OR (son[TIAB]) OR (daughter[TIAB]) OR (boy[TIAB]) OR (girl[TIAB]) OR (offspring[TIAB])) AND ((relation*[TIAB]) OR (interact*[TIAB]) OR (communicat*[TIAB]))) |
| #4 | #1 AND #2 AND #3 |

PsycINFO (EBSCOhost)

| Number | Query |
| --- | --- |
| S1 | DE "Parents" OR DE "Mothers" OR DE "Fathers" OR TI ("parent*" OR "mother*" OR "father*" OR "caregiver*") OR AB ("parent*" OR "mother*" OR "father*" OR "caregiver*") |
| S2 | MM "Emotional Regulation" OR TI ("emotion* regulat*" OR "emotion* dysregulat*" OR "affect* regulat*" OR "affect* dysregulat*") OR AB ("emotion* regulat*" OR "emotion* dysregulat*" OR "affect* regulat*" OR "affect* dysregulat*") |
| S3 | MM "Parenting" OR MM "Authoritarian Parenting" OR MM "Authoritative Parenting" OR MM "Parent Child Communication" OR MM "Parent Child Relations" OR MM "Parental Involvement" OR MM "Parenting Skills" OR MM "Parenting Style" OR MM "Permissive Parenting" OR MM "Child Abuse" OR MM "Child Neglect" OR TI (parenting OR "parent* behavi*" OR "maternal behavi*" OR "paternal behavi*" OR "parent* discipline practice*" OR "parent* negativity" OR "maternal negativity" OR "paternal negativity" OR "parent* warmth" OR "maternal warmth" OR "paternal warmth" OR "parent* control" OR "maternal control" OR "paternal control" OR "parent* hostility" OR "maternal hostility" OR "paternal hostility" OR "parent* sensitivity" OR "maternal sensitivity" OR "paternal sensitivity" OR "emotion* sociali#ation" OR "child abus*" OR "child neglect*" OR "child maltreat*") OR  AB (parenting OR "parent* behavi*" OR "maternal behavi*" OR "paternal behavi*" OR "parent* discipline practice*" OR "parent* negativity" OR "maternal negativity" OR "paternal negativity" OR "parent* warmth" OR "maternal warmth" OR "paternal warmth" OR "parent* control" OR "maternal control" OR "paternal control" OR "parent* hostility" OR "maternal hostility" OR "paternal hostility" OR "parent* sensitivity" OR "maternal sensitivity" OR "paternal sensitivity" OR "emotion* sociali#ation" OR "child abus*" OR "child neglect*" OR "child maltreat*") OR TI ((parent OR mother OR father) AND (child OR infant OR baby OR toddler OR adolescent OR son OR daughter OR boy OR girl) AND (relation* OR interact* OR communicat*)) OR AB ((parent OR mother OR father) AND (child OR infant OR baby OR toddler OR adolescent OR son OR daughter OR boy OR girl OR offspring) AND (relation* OR interact* OR communicat*)) |
| S4 | S1 AND S2 AND S3 |

Embase (Ovid)

| Number | Query |
| --- | --- |
| 1 | parent/ or mother/ or father/ or caregiver/ or (parent$ or mother$ or father$ or caregiver$).ti,ab,kw. |
| 2 | exp *self control/ or (emotion$ regulat$ or emotion$ dysregulat$ or affect$ regulat$ or affect$ dysregulat$).ti,ab,kw. |
| 3 | exp *parental behavior/ or exp *child parent relation/ or child abuse/ or child neglect/ or (parenting or parent$ behavi$ or maternal behavi$ or paternal behavi$ or parent$ discipline practice$ or parent$ negativity or maternal negativity or paternal negativity or parent$ warmth or maternal warmth or paternal warmth or parent$ control or maternal control or paternal control or parent$ hostility or maternal hostility or paternal hostility or parent$ sensitivity or maternal sensitivity or paternal sensitivity or emotion$ socialization or emotion$ socialization or child abus$ or child neglect$ or child maltreat$).ti,ab,kw. or ((parent or mother or father) and (child or infant or toddler or baby adolescent or son or daughter or boy or girl or offspring) and (interact$ or relation$ or communicat$)).ti,ab,kw. |
| 4 | 1 and 2 and 3 |
| 5 | limit 4 to exclude medline journals |

Web of Science (Core Collection)

| Number | Query |
| --- | --- |
| #1 | TS=(parent* OR mother* OR father* OR caregiver*) |
| #2 | TS=("emotion* regulat*" OR "affect* regulat*" OR "emotion* dysregulat*" OR "affect* dysregulat*") |
| #3 | TS=(parenting OR "parent* behavi*" OR "maternal behavi*" OR "paternal behavi*" OR "parent* discipline practice*" OR "parent* negativity" OR "maternal negativity" OR "paternal negativity" OR "parent* warmth" OR "maternal warmth" OR "paternal warmth" OR "parent* control" OR "maternal control" OR "paternal control" OR "parent* hostility" OR "maternal hostility" OR "paternal hostility" OR "parent* sensitivity" OR "maternal sensitivity" OR "paternal sensitivity" OR "emotion* socialization" OR "emotion* socialisation" OR "child abus*" OR "child neglect*" OR "child maltreat*") OR (TS=(parent OR mother OR father) AND TS=(child OR infant OR toddler OR baby OR adolescent OR son OR daughter OR boy OR girl OR offspring) AND TS=(relation* OR interact* OR communicat*)) |
| #4 | #3 AND #2 AND #1 |

**Appendix B: Extraction protocol**

General information:

- Study ID
- Title
- Year
- Authors
- Lead author contact details
- Journal
- DOI
- Country (study conducted)
- Main aims
- Secondary aims
- Study design
  - Cross sectional
  - Case control
  - Cohort
- Start date (data collection)
- End date (data collection)
- Study funding sources
- Possible conflicts of interest
- Notes

Sample:

- Focus of recruitment
  - Parents
  - Children
- Sample
  - Mothers
  - Fathers
  - Both parents/ either mothers or fathers
  - Others
- Setting (parent or children)
  - Clinical: Inpatient
  - Clinical: Outpatient
  - Non-clinical/ Community sample
  - Other
- Method of recruitment of participants
- Research question/ hypothesis
- Total number of participants
- Comparison groups
  - Clinical
  - Healthy
  - No
  - Other
- Notes

Group 1:

- Name of group
- Total number of participants
- Sex parents
- Age parents
- Education
- Socioeconomic status
- Psychopathology
  - Mental Disorder
  - Psychopathology at a clinical level
  - Psychopathology at a subclinical level
- Type of psychopathology
- Inclusion criteria
- Exclusion criteria
- Comorbidities
- Notes

Group 2:

- Name of group
- Total number of participants
- Sex parents
- Age parents
- Education
- Socioeconomic status
- Psychopathology
  - Mental Disorder
  - Psychopathology at a clinical level
  - Psychopathology at a subclinical level
- Type of psychopathology
- Inclusion criteria
- Exclusion criteria
- Comorbidities
- Notes

Measures:

- Measure(s) of psychopathology
- Measure(s) of emotion (dys)regulation
- Measure(s) of parenting
- Other measure(s)

Results:

- Data analysis plan
- Number of participants analyzed
- Number of participants excluded
- Reason for exclusion
- Missing values
- Main Results relevant to review questions (including statistical values)
- Other Results (including statistical values)

Discussion:

- Main conclusion relevant to review questions
- Other conclusions
- Reference to other relevant studies
- Limitations
- Notes

**Appendix C: Quality assessment of the included studies**

Adopted Version of the Joanna Briggs Institute (JBI) Critical Appraisal Checklist for analytical Cross-Sectional Study (Moola et al., 2020)

| First author (Year) | 1. Were the criteria for inclusion in the sample clearly defined? | 2. Were the study subjects and the setting described in detail? | 3. Was emotion regulation measured in a valid and reliable way? | 4. Were objective, standard criteria used for measure-ment of psycho-pathology? | 5. Were confounding factors identified? | 6. Were strategies to deal with confounding factors stated? | 7. Was parenting measured in a valid and reliable way? | 8. Was appropriate statistical analysis used? | Overall appraisal |
| --- | --- | --- | --- | --- | --- | --- | --- | --- | --- |
| Bao (2020) | NA | Y | Y | Y | Y | Y | Y | Y | Y |
| Bertie (2021) | Y | Y | Y | Y | Y | Y | Y | Y | Y |
| Brake (2020) | Y | Y | Y | Y | NA | NA | Y | Y | Y |
| Caçador (2021) | Y | Y | Y | Y | Y | Y | Y | Y | Y |
| Casline (2020) | Y | Y | Y | Y | Y | Y | Y | Y | Y |
| Dittrich (2018) | Y | Y | Y | Y | Y | Y | Y | Y | Y |
| Doba (2022) | NA | Y | Y | Y | Y | Y | Y | Y | Y |
| Gurtovenko (2020) | Y | Y | Y | Y | NA | NA | Y | Y | Y |
| Hien (2010) | Y | Y | Y | Y | Y | Y | Y | Y | Y |
| Kiel (2017) | Y | Y | Y | Y | Y | Y | Y | Y | Y |
| Kohlhoff (2016) | Y | Y | Y | Y | Y | Y | Y | Y | Y |
| Kumar (2019) | Y | Y | Y | Y | Y | Y | Y | Y | Y |
| Lotzin (2015) | Y | Y | Y | Y | Y | Y | Y | Y | Y |
| Lotzin (2016) | Y | Y | Y | Y | Y | Y | Y | Y | Y |
| McCurdy (2022) | NA | Y | Y | Y | NA | NA | Y | Y | Y |
| Powers (2021) | Y | Y | Y | Y | Y | NA | Y | Y | Y |
| Raveau (2019) | Y | Y | Y | Y | Y | Y | Y | Y | Y |

*Notes.* Y = yes, N = no, NA = not applicable.

Adopted Version of the Joanna Briggs Institute (JBI) Critical Appraisal Checklist for Cohort Studies (Moola et al., 2020)

| First author (Year) | 1. Were all partici-pants recruited from the same population? | 2. Was emotion regulation measured in the same way for all partici-pants? | 3. Was emotion regulation measured in a valid and reliable way? | 4. Were confound-ding factors identified? | 5. Were strategies to deal with confounding factors stated? | 6. Was the exposure assessed before the outcome? | 7. Was parenting measured in a valid and reliable way? | 8. Was the follow up time reported and sufficient to be long enough for outcomes to occur? | 9. Was follow up complete, and if not, were the reasons to loss to follow up described and explored? | 10. Were strategies to address income-plete follow up utilized? | 11. Was appropri-ate statistical analysis used? | Overall appraisal |
| --- | --- | --- | --- | --- | --- | --- | --- | --- | --- | --- | --- | --- |
| Behrendt (2019) | Y | Y | Y | Y | NA | Y | Y | NA | NA | NA | Y | Y |
| Jensen (2021) | Y | Y | Y | Y | NA | Y | Y | Y | Y | NA | Y | Y |
| Mazursky-Horowitz (2015) | Y | Y | Y | Y | NA | Y | Y | Y | Y | Y | Y | Y |
| Price (2021) | Y | Y | Y | Y | Y | Y | Y | Y | Y | N | Y | Y |

*Notes.* Y = yes, N = no, NA = not applicable.

Adopted Version of the Joanna Briggs Institute (JBI) Critical Appraisal Checklist for Case-Control Studies (Moola et al., 2020)

| First author (Year) | 1. Were the groups comparable regarding relevant study variables other than the presence of parenting difficulties in cases or the absence of parenting difficulties in controls? | 2. Were cases and controls matched appropri-ately? | 3. Were the same criteria used for identify-cation of cases and controls? | 4. Was emotion regulation measured in a valid and reliable way? | 5. Was emotion regulation measured in the same way for cases and controls? | 6. Were confound-ding factors identified? | 7. Were strategies to deal with confound-ding factors stated? | 8. Was parenting measured in a valid and reliable way? | 9. Was the exposure period (emotion regulation) of interest long enough to be meaningful? | 10. Was appropriate statistical analysis used? | Overall appraisal |
| --- | --- | --- | --- | --- | --- | --- | --- | --- | --- | --- | --- |
| Hiraoka (2016) | Y | N | Y | Y | Y | Y | Y | Y | NA | Y | Y |
| Stover (2013) | NA | NA | Y | Y | Y | Y | Y | Y | NA | Y | Y |

*Notes.* Y = yes, N = no, NA = not applicable.
